# MuCHLoc: Indoor ZigBee Localization System Utilizing Inter-Channel Characteristics †

**DOI:** 10.3390/s19071645

**Published:** 2019-04-06

**Authors:** Ryota Kimoto, Shigemi Ishida, Takahiro Yamamoto, Shigeaki Tagashira, Akira Fukuda

**Affiliations:** 1 Graduate School/Faculty of Information Science and Electrical Engineering, Kyushu University, Fukuoka 819-0395, Japan; ishida@f.ait.kyushu-u.ac.jp (S.I.); yamamoto@f.ait.kyushu-u.ac.jp (T.Y.); fukuda@f.ait.kyushu-u.ac.jp (A.F.); 2 Faculty of Informatics, Kansai University, Osaka 569-1095, Japan; shige@res.kutc.kansai-u.ac.jp

**Keywords:** ZigBee sensor localization, fingerprinting, cross-technology signal detection, multi-channel, channel diversity, frequency selective fading

## Abstract

The deployment of a large-scale indoor sensor network faces a sensor localization problem because we need to manually locate significantly large numbers of sensors when Global Positioning System (GPS) is unavailable in an indoor environment. Fingerprinting localization is a popular indoor localization method relying on the received signal strength (RSS) of radio signals, which helps to solve the sensor localization problem. However, fingerprinting suffers from low accuracy because of an RSS instability, particularly in sensor localization, owing to low-power ZigBee modules used on sensor nodes. In this paper, we present MuCHLoc, a fingerprinting sensor localization system that improves the localization accuracy by utilizing channel diversity. The key idea of MuCHLoc is the extraction of channel diversity from the RSS of Wi-Fi access points (APs) measured on multiple ZigBee channels through fingerprinting localization. MuCHLoc overcomes the RSS instability by increasing the dimensions of the fingerprints using channel diversity. We conducted experiments collecting the RSS of Wi-Fi APs in a practical environment while switching the ZigBee channels, and evaluated the localization accuracy. The evaluations revealed that MuCHLoc improves the localization accuracy by approximately 15% compared to localization using a single channel. We also showed that MuCHLoc is effective in a dynamic radio environment where the radio propagation channel is unstable from the movement of objects including humans.

## 1. Introduction

Recent advances in wireless communication and computing technologies have resulted in successful low-cost low-power wireless sensor networks (WSNs), particularly in the fields of cyber physical systems (CPSs) and the Internet of Things (IoT). In a WSN, sensor location is important information for recognizing and tracking a sensing target, and for the routing of a sensor network. In an outdoor environment, we can locate sensor nodes using Global Positioning System (GPS). In indoor environments, we need to manually measure the sensor nodes. When we build a large-scale sensor network, we need to locate an extremely large number of sensor nodes.

Indoor sensor localization systems have been proposed to reduce the efforts regarding sensor localization in an indoor environment [1,2,3]. Studies have primarily focused on improved accuracy [4,5,6,7,8,9]. Fingerprinting localization is a popular localization method using the received signal strength (RSS) owing to its high accuracy [10,11,12,13,14,15,16,17,18].

However, fingerprinting still suffers from low accuracy when the RSS is unstable owing to an unstable radio propagation environment. In particular, sensor nodes equipped with ZigBee (IEEE 802.15.4) modules transmit radio signals with considerably lower power compared to Wi-Fi (IEEE 802.11) modules. In addition, ZigBee uses narrow-band channels, which are significantly affected by frequency selective fading. When a ZigBee module measures the RSS of wide-band radio signals from an identical signal source, the RSS values are dependent on the measurement ZigBee channels. In an indoor environment, i.e., a dynamic and multipath environment, the measured RSS becomes more unstable compared to Wi-Fi. The localization accuracy deteriorates from an unstable RSS.

This paper presents *MuCHLoc*, a fingerprinting sensor localization system that improves the localization accuracy by utilizing channel diversity. The key idea of MuCHLoc is to extract channel diversity from the RSS of Wi-Fi access points (APs) measured on multiple ZigBee channels during fingerprinting localization. The RSS is dependent on the measured channel and location because narrow-band ZigBee is highly affected by frequency selective fading. We collect the RSS of Wi-Fi APs measured on multiple ZigBee channels and construct a fingerprint database, which includes channel diversity information. MuCHLoc overcomes the instability of the RSS by increasing the dimensions of the fingerprints using channel diversity.

MuCHLoc measures the RSS of wide-band Wi-Fi AP signals sent from Wi-Fi APs while switching the ZigBee channels. Wi-Fi is a wide-band communication technology whose channel overlaps with the four ZigBee channels. We can measure the RSS on the four ZigBee channels without switching the Wi-Fi transmission channel. Using Wi-Fi APs previously installed in an environment, no newly deployed reference nodes are required. ZigBee sensor nodes cannot demodulate Wi-Fi signals. We therefore utilize a multi-channel cross-technology signal detection method.

We conducted experiments to collect RSS samples at our university laboratory and analyzed the samples to evaluate the performance of MuCHLoc. We conducted a 10-fold leave-one-out cross validation with 1000 RSS samples collected at five locations to estimate the location of the sensor nodes, evaluating the localization accuracy. The experimental evaluation revealed that MuCHLoc improves the localization accuracy by approximately 15% compared to the localization system relying on the RSS of the Wi-Fi AP measured on one ZigBee channel. We also demonstrate that MuCHLoc is effective in a dynamic environment where the radio propagation channel is unstable.

MuCHLoc is an extension of *MultiZigLoc* presented in our conference paper [19]. We conducted additional evaluations to validate the effectiveness of our proposed method. The additional evaluation described in Section 4.3 validates the channel diversity, which is a key to MuCHLoc. We also describe another additional evaluation in Section 4.5 to validate the effectiveness of MuCHLoc over an unstable RSS caused by a dynamic environment.

Specifically, our main contributions are threefold:

- We present the design of MuCHLoc, a fingerprinting sensor localization system utilizing channel diversity. MuCHLoc measures the RSS of Wi-Fi APs on multiple ZigBee channels and extracts channel-specific features at the location to improve the localization accuracy. To the best of our knowledge, this is the first attempt at developing a sensor localization system employing cross-technology multi-channel RSS measurements deriving the channel diversity.
- We evaluate MuCHLoc using sensor nodes and Wi-Fi APs deployed in a practical environment. We demonstrate that MuCHLoc improves the localization accuracy compared to localization without channel diversity.
- We experimentally confirm that MuCHLoc is effective in a dynamic radio environment where the radio propagation channel is unstable owing to the presence of moving objects including humans.

The remainder of this paper is organized as follows. In Section 2, we summarize related studies on indoor sensor localization including an accuracy improvement utilizing channel diversity. The design of MuCHLoc is presented in Section 3, followed by experimental evaluations validating the effectiveness of MuCHLoc in Section 4. Finally, Section 5 provides some concluding remarks regarding this research.

## 2. Related Work

To the best of our knowledge, this is the first paper reporting a ZigBee sensor localization system employing channel diversity derived from the RSS of Wi-Fi APs measured using a cross-technology signal detection method. This study is related to (i) radio-based indoor sensor localization and (ii) accuracy improvements utilizing channel diversity.

### 2.1. Indoor Sensor Localization

Radio-based indoor localization methods are categorized into range-based and range-free methods.

Range-based localization methods first estimate the range, i.e., distance, between the target and reference nodes, and then estimate the target location using a geometrical approach such as multilateration. Range-based localization methods also require reference nodes whose locations are known prior to localization. The distance between the target and reference nodes is estimated using a radio path-loss model with the RSS of the radio signals measured during radio communication between the target and reference nodes.

For range-based localization, previous studies have mainly tried to reduce the deployment costs. Iterative multilateration [20] is a popular method in which localized target nodes are used as new reference nodes. Iterative multilateration reduces the number of reference nodes during the initial setup. The number of references can also be reduced through reference location optimization [21]. These methods can be combined with our MuCHLoc to locate sensor nodes with a small number of Wi-Fi APs.

Range-free localization relies on non-physical information such as the network connectivity. Range-free localization has been well studied for use in a WSN and an ad hoc network where limited computational resources are available. Centroid [22], DV-Hop [23,24], Amorphous [8], and APIT [25,26] are famous pioneering range-free localization methods. These pioneering localization methods require small computational resources but suffer from low localization accuracy owing to the small correlation between the network connectivity and geometric information.

Fingerprinting localization [9] is also a range-free localization and is widely in use today. Fingerprinting localization is a machine-learning based localization algorithm that consists of training and localization phases. The training phase conducts a site-survey for collecting fingerprints, i.e., vectors consisting of the RSS of multiple reference nodes, at many points in a target area, and constructs a fingerprint database. A fingerprint in the fingerprint database is a feature vector that represents the location where the fingerprint is collected. During the localization phase, a target sensor node measures the RSS of radio signals sent from the reference nodes and creates a target fingerprint. Sensor location is estimated by finding the database fingerprint closest to the target fingerprint.

However, fingerprinting suffers from low accuracy when the RSS is unstable owing to unstable radio propagation environments. In an indoor environment, i.e., a dynamic and multipath environment, the ZigBee RSS becomes more unstable compared to Wi-Fi because of a narrower band, which is highly affected by multipath fading. ZiFind and ZIL presented new learning algorithms to address the multipath problem [15,16]. We also presented a sensor localization system utilizing the channel difference [19]. This paper extends our conference paper [19] to validate the effectiveness of our method in a more practical environment.

Fingerprinting localization requires extremely large numbers of fingerprints for accurate localization. Studies have therefore been conducted to reduce the site-survey effort required [10,11,12,13]. ZigLoc is a ZigBee-based sensor localization system relying on the RSS of Wi-Fi APs as a reference derived through cross-technology communication [17,18]. ZigLoc uses Wi-Fi fingerprints instead of fingerprints collected by sensor nodes, removing the requirement of an additional site-survey. A Wi-Fi fingerprint is a feature vector consisting of an RSS of Wi-Fi APs measured using Wi-Fi devices, and is available when a Wi-Fi fingerprinting localization system is already in use. These technologies, which can be combined with MuCHLoc without collisions, are also useful for a site-survey cost reduction.

### 2.2. Accuracy Improvement Using Channel Diversity

Channel diversity has been utilized in certain studies to improve the localization accuracy. Such studies are mainly based on orthogonal frequency division multiplexing (OFDM), which is a type of modulation used in Wi-Fi. OFDM uses multiple carrier waves called subcarriers. Channel state information (CSI) fingerprinting [27] employs radio propagation characteristics of the subcarriers as location-specific features in fingerprinting localization. Splicer [28] and Chronos [29] virtually combine Wi-Fi channels to precisely estimate the time of flight of the Wi-Fi signals, which is used in range-based localization. Sensor nodes, however, use ZigBee communication whose modulation is not OFDM.

MCRA [30] combines multiple ZigBee channels to improve ZigBee-based ranging accuracy. MCRA calculates the RSS averaged over multiple ZigBee channels to derive a stable RSS. Separate channel fingerprinting is a Bluetooth Low Energy (BLE) localization system utilizing channel diversity [31]. These approaches implicitly assume that the transmitter and receiver are the same wireless technology devices. MCRA, for example, measures the RSS while switching the ZigBee channels synchronously at both the transmitter and receiver. The synchronization requires time and energy to exchange synchronization control packets between the transmitter and receiver. Localization requires the RSS of multiple reference nodes, which results in a large time/energy overhead in addition to RSS measurements on multiple channels.

MuCHLoc differs from these studies in that MuCHLoc measures the RSS of incompatible wide-band Wi-Fi signals using a ZigBee sensor node. No channel switching is required on the transmitter side. We can utilize Wi-Fi APs already installed in the environment.

## 3. MuCHLoc

### 3.1. Key Idea

**Figure 1:**
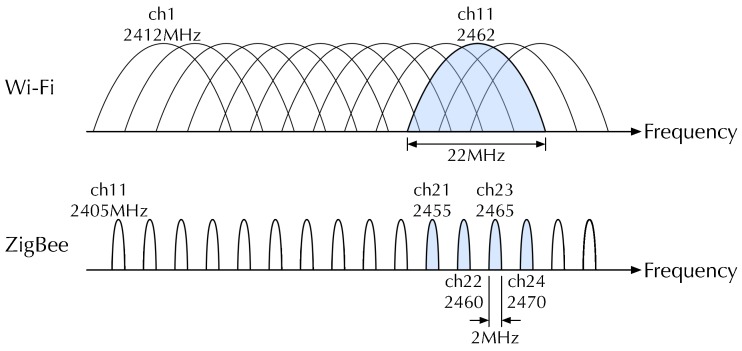
Channel coordination of Wi-Fi and ZigBee.

The key idea of MuCHLoc is that we use channel diversity relying on wide-band Wi-Fi signals to improve the ZigBee localization accuracy. Figure 1 shows the channel coordination of Wi-Fi and ZigBee. As the figure indicates, a Wi-Fi channel and four ZigBee channels share the same frequency band. For example, Wi-Fi channel 11 and ZigBee channels 21–24 use the frequency band from 2451 to 2473 MHz. We measure the Wi-Fi AP-RSS of an identical AP on the four overlapping ZigBee channels to derive the inter-channel characteristics. Wi-Fi signals are wide-band, and no Wi-Fi channel switching is required for RSS measurements on multiple ZigBee channels. We can utilize Wi-Fi APs already installed in the environment.

Although its key idea is quite simple, a ZigBee device cannot demodulate Wi-Fi signals because ZigBee and Wi-Fi are completely different wireless technologies. We therefore employ a multi-channel cross-technology signal detection method [32].

### 3.2. Design Overview

**Figure 2:**
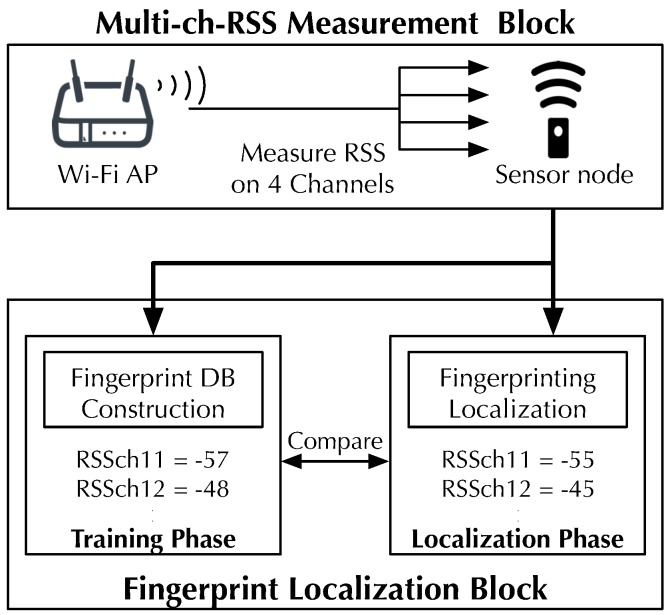
Overview of MuCHLoc.

Figure 2 shows a design overview of MuCHLoc, which consists of two blocks: multi-ch-RSS measurement and fingerprint localization blocks. The multi-ch-RSS measurement block measures the Wi-Fi AP-RSS using a cross-technology signal detection method while changing the measurement ZigBee channel within the four channels overlapping a Wi-Fi channel. In the fingerprint localization block, sensor location is estimated using a fingerprinting method with the AP-RSS measured on four ZigBee channels.

In the following subsections, the design details of each block are presented.

### 3.3. Multi-ch-RSS Measurement Block

The multi-ch-RSS measurement block consists of Wi-Fi APs and a ZigBee sensor node. Wi-Fi APs send beacon signals periodically, as defined in the standard. A ZigBee sensor node detects the AP beacon signals and measures their RSS. The sensor node repeats the RSS measurement on four channels overlapping with a Wi-Fi AP operating channel.

Wi-Fi beacon signals cannot be directly detected on a ZigBee sensor node. A ZigBee sensor node conducts simple signal processing based on the periodicity of beacons to detect the AP beacon signals. Although ZigBee and Wi-Fi use an incompatible modulation, they are on the same 2.4-GHz band. The Wi-Fi signals are detected using ZigBee node with its RSS measurement capability defined in the standard [33].

**Figure 3:**
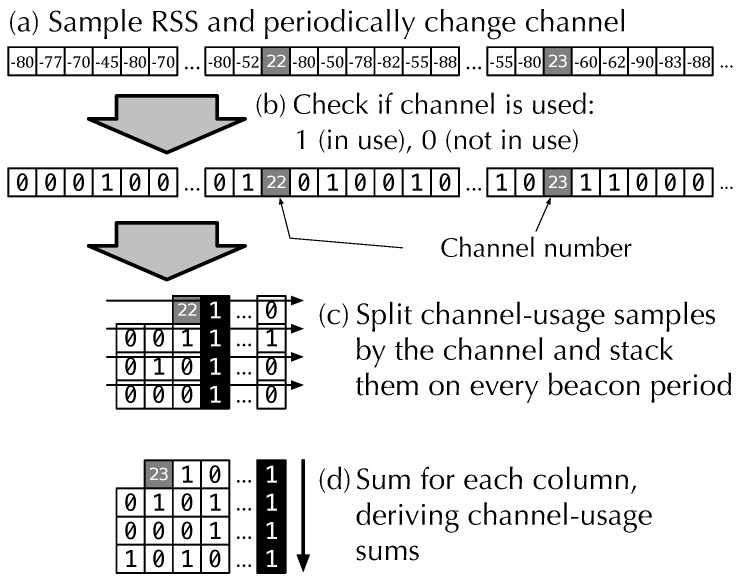
Overview of multi channel AP signal detection in multi-ch-RSS measurement block.

Figure 3 shows an overview of the AP beacon detection. A sensor node continuously measures the RSS of any radio signals on a ZigBee channel at a specific interval (Figure 3a). The standard defines the RSS measurement capability to provide the RSS averaged over a period of 128 ms. The RSS sampling rate is set to every 128 ms to avoid a time blank between the RSS samples with the minimum sampling rate.

ZigBee channel is changed when specific numbers of RSS samples are collected. A channel change requires a radio circuit restart. We store the channel number instead of the RSS during the circuit restart.

Based on the RSS samples, we check if the channel is being used. The channel-usage samples describe whether the channel is *in use* (1) or *not in use* (0), as shown in Figure 3b. The threshold for the channel usage is set to −77 dBm, which is a default threshold of a clear channel assessment on a CC2420 IEEE 802.15.4 module [34].

The channel-usage samples are divided by the measurement channel and are stacked during every beacon period, resulting in channel-usage matrices (Figure 3c). The channel-usage matrices might be incomplete, as shown in Figure 3, because the number of RSS samples might not equal the multiples of the beacon period. Finally, the sums, which we call channel-usage sums, for each column are calculated for each channel-usage matrix (Figure 3d).

The multi-ch-RSS measurement block finds columns whose channel-usage sum is above a specific threshold. Periodic beacon signals appear on a specific column because the beacon interval equals the stacking period.

We average RSS samples corresponding to the columns above the threshold on a channel-usage matrix to derive the AP-RSS. A simple edge filter used to minimize the RSS measurement error caused by a partial RSS problem [35] is also employed. When all channel-usage sums are smaller than 70% of the number of stacks, which follows after the threshold in [35], we regard the beacon signal to be missing. The RSS of the missing beacons is marked as missing.

The RSS measurement process takes a long time because it requires channel-usage matrices having 30 or more rows to detect periodic beacon signals. One row of the channel-usage matrix corresponds to the beacon period. The beacon period is set to 100 TU on many Wi-Fi APs, which implies that the RSS measurement on one ZigBee channel takes approximately 3 s. We believe that we can drastically reduce the RSS measurement time using recent cross-technology communication technologies such as [36].

When we measure the RSS of multiple APs, each AP is configured with non-multiple beacon intervals to distinguish the sender APs [37]. The multi-ch-RSS measurement block stacks channel-usage samples on a beacon interval. Beacon signals with a non-multiple interval of the stacking period are not detected in a channel-usage matrix. We repeat the stacking process for each beacon interval to separately detect the APs.

### 3.4. Fingerprint Localization Block

**Figure 4:**
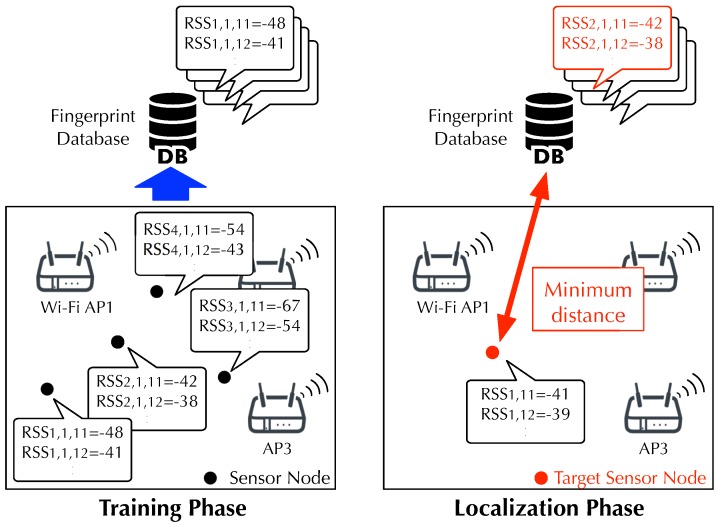
Overview of fingerprint localization block.

Figure 4 shows an overview of the fingerprinting localization block. The fingerprinting localization block estimates the sensor location using a fingerprinting method. Fingerprinting in MuCHLoc consists of training and localization phases, similar to normal fingerprinting.

During the training phase, the fingerprinting localization block conducts a site-survey to construct a fingerprint database for storing fingerprints. A fingerprint is a feature vector consisting of the RSS of multiple APs, which is used for machine-learning based fingerprinting localization. We collect the RSS on multiple ZigBee channels at multiple locations in a target area and calculate a database fingerprint vector $R_{i}$ for each location *i* as follows:

(1) $$R_{i}=\left\{r_{i,1,21},r_{i,1,22},\ldots ,r_{i,2,21},\ldots ,r_{i,n,c}\right\},$$

where $r_{i,j,c}$ is the average RSS of ${\mathrm{AP}}_{j}$ measured on a ZigBee channel *c* at location *i*.

Because MuCHLoc utilizes location-specific fingerprints, the fingerprint database needs to be updated when the placement of the objects is drastically changed. We can utilize existing fingerprint updating methods such as Chameleon [38] and LAAFU [39].

During the localization phase, the sensor location is estimated using the same procedure as a normal fingerprinting localization method. The fingerprinting localization block finds a fingerprint in the database closest to the target fingerprint consisting of the RSS collected through a localization target node. A target fingerprint $\bar{t}$ is defined in the same manner as in Equation (1) without location *i*:

(2) $$\bar{t}=\left\{t_{1,21},t_{1,22},\ldots ,t_{2,21},\ldots ,t_{n,c}\right\},$$

where $t_{j,c}$ is the average RSS of ${\mathrm{AP}}_{j}$ measured on ZigBee channel *c*. We calculate the similarity between the database and target fingerprints using the distance based on the root-mean-square as follows:

(3) $$\begin{array}{cc} \mathrm{dist}(R_{i},\bar{t}) & =\sqrt{\frac{1}{|\bar{t}|}\sum_{n,c}{\left(r_{i,n,c}-t_{n,c}\right)}^{2}}, \end{array}$$

where $|\bar{t}|$ is the number of non-missing elements in both $R_{i}$ and $\bar{t}$. We ignore missing elements, i.e., the RSS of undetected APs.

To reduce the influence of the RSS instability, we apply a moving average filter to target fingerprints derived as a function of time. Let $\bar{t[k]}$ be a target fingerprint at time index *k*. The averaged target fingerprint $\tilde{t[k]}$ at time index *k* is defined as follows:

(4) $$\tilde{t[k]}=\sum_{l=k-w+1}^{k}\bar{t[l]},$$

where *w* is the window size of the moving average. Note that we ignore target fingerprints such that more than one half of the elements are marked as missing.

Finally, the sensor location is estimated at each time index *k* using a nearest neighbor method. The target location $\hat{ı}$ is estimated by finding a database fingerprint $R_{i}$ having the minimum distance to the target fingerprint $\tilde{t[k]}$:

(5) $$\hat{ı}=\underset{i}{arg min}\mathrm{dist}\left(R_{i},\tilde{t[k]}\right).$$

## 4. Evaluation

We implemented the MuCHLoc and conducted experiments to evaluate the localization performance.

### 4.1. Implementation

**Figure 5:**
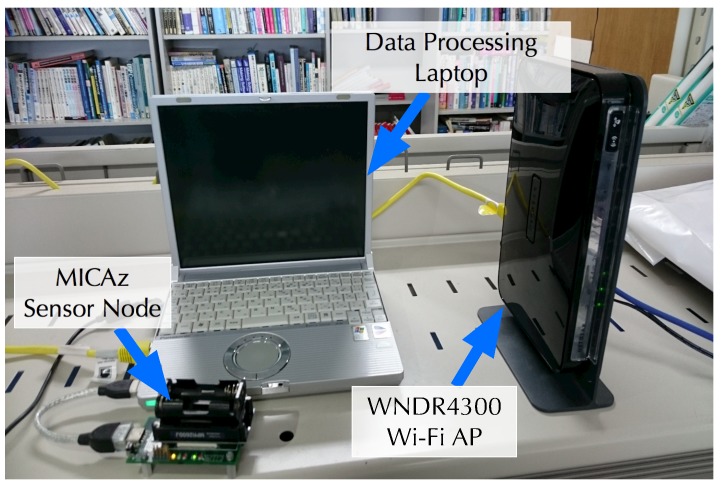
Equipment used in our experimental evaluations.

Figure 5 shows the equipment used in our experimental evaluations. As the Wi-Fi APs, Netgear WNDR4300 APs running OpenWrt, which is a Linux-based OS (operating system) for access points, were utilized. The sensor node was a Crossbow MICAz employing a CC2420 IEEE 802.15.4 module (Milpitas, CA, USA) [34]. Our entire system was implemented through Python programs on Debian/GNU Linux 9.5 (Software in the Public Interest, Inc., New York, NY, USA) running on a Panasonic CF-R4 data processing laptop computer (Osaka, Japan).

The sensor node was controlled using a data processing laptop to sample the RSS on a specific ZigBee channel. The RSS samples were sent to the data processing laptop, where the multi-ch-RSS measurement block applied the signal processing described in Section 3.3 to measure the AP-RSS on multiple ZigBee channels. The fingerprinting localization described in Section 3.4 was finally applied to estimate the sensor location.

### 4.2. Experiment Environment

**Figure 6:**
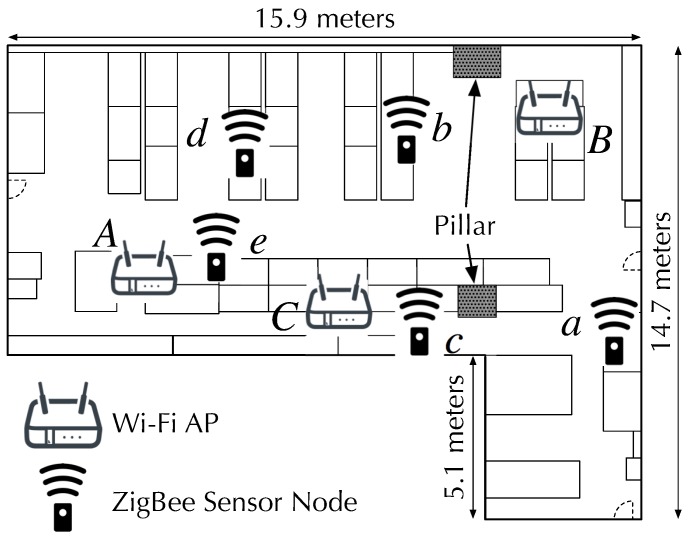
Experiment environment in our laboratory.

Figure 6 shows the experiment environment. The experiment environment was a complicated multipath environment. There were many metallic desks with partitions and metallic shelves in addition to concrete pillars, which all highly affect the Wi-Fi signal propagation. For our experiment, we configured existing Wi-Fi APs *A*, *B*, and *C* in our laboratory. We collected AP-RSS data on three APs *A*, *B*, and *C* at five locations labeled *a*, *b*, *c*, *d*, and *e*. We configured the APs using beacon intervals of 101, 103, and 107 TU to separately measure the RSS of each AP. Wi-Fi APs were configured to operate on channel 11. We should note that more than ten Wi-Fi APs, which were not used in our experiment, with a beacon interval of 100 TU were in use for communication within our experiment environment.

AP-RSS were measured on ZigBee channels 21–24 corresponding to Wi-Fi channel 11. On each ZigBee channel, we collected RSS samples for 4 s and repeated the RSS collection 1000 times. Trials in which the AP signals were detected on less than two ZigBee channels were excluded in our evaluation.

We conducted three evaluations:

- We validated two key observations upon which MuCHLoc relies as the preliminary experiments. First, we validated the RSS instability, which implies the difficulty of indoor localization in our environment. We next validated the channel diversity, which is a key observation in MuCHLoc to improve the localization accuracy. We applied Welch’s two-sample *t*-tests to confirm the RSS difference on different ZigBee channels at different locations.
- We validated the localization accuracy of MuCHLoc using a 10-fold leave-one-out cross validation. We repeated the cross validation 100 times with shuffled data to estimate the sensor location within the five labeled locations and evaluated the localization accuracy.
- We validated the effectiveness of MuCHLoc in a dynamic radio propagation environment. We compared the localization accuracy between dynamic and static environments.
- We validated the localization accuracy of MuCHLoc using multiple APs. The RSS samples from three APs were collected. We then evaluated the localization accuracy using RSS samples from multiple APs in the same way as with a single AP.

**Figure 7:**
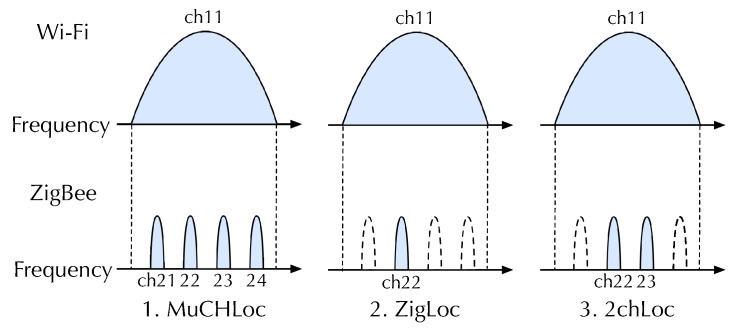
ZigBee channels used in MuCHLoc, ZigLoc and 2chLoc localization.

For the localization accuracy evaluations, we compared the localization accuracy of three fingerprinting localization methods listed below to evaluate their relative performance. Figure 7 conceptually illustrates the differences among the three methods:

1. MuCHLoc: MuCHLoc is the proposed method described in Section 3, which utilizes channel diversity derived from the RSS of wide-band Wi-Fi AP signals during fingerprinting.
2. ZigLoc [17,18]: ZigLoc is a fingerprinting method utilizing Wi-Fi AP-RSS without channel diversity. AP-RSS is measured on a single ZigBee channel overlapping a Wi-Fi AP operating channel.
3. 2chLoc: 2chLoc is a fingerprinting method utilizing channel diversity on a limited number of channels. AP-RSS is measured on two ZigBee channels overlapping at the center of a Wi-Fi AP operating channel.

### 4.3. Preliminary Experiments

We validated two key observations upon which MuCHLoc relies, i.e., RSS instability and channel diversity, as preliminary experiments.

**Figure 8:**
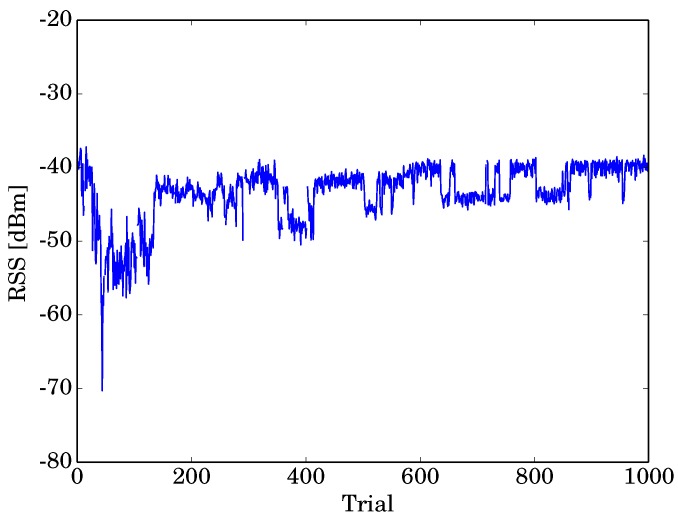
RSS of AP *B*, measured by sensor node *e*, as a function of time.

First, we validated the RSS instability, which implies the difficulty of localization in our experiment environment. Figure 8 shows the RSS of the AP *B*, measured using the sensor node *e* in a dynamic environment, as a function of time. The maximum and minimum values of the RSS were −37.2 dBm and −70.3 dBm, respectively. We can confirm that the RSS was unstable because of a strong multipath effect.

We next validated the difference in RSS on different channels and at different locations because MuCHLoc utilizes the RSS depending on the location and channel as the channel diversity. To confirm the difference in RSS on different ZigBee channels at different locations, we applied Welch’s *t*-tests on the RSS samples. Welch’s *t*-tests confirm whether the averages of two distributions are the same. We checked the difference between the distributions of the RSSs at different locations and on different channels.

Table 1 and Table 2 show the *p*-values of the Welch’s two-sample *t*-tests on the RSSs measured at different locations and on different channels, respectively. Note that not all location/channel combinations are presented in the tables. The columns and rows of the tables correspond to the location/channel at/upon which the RSS samples were collected. In Table 1 and Table 2, an extremely small number of cells are non-zero, which are highlighted in the tables. At a significance level of p<0.05, we confirmed that the RSS was significantly different for all location/channel combinations. We also confirmed the same results for all other combinations of location/channel that are not presented in the tables.

### 4.4. Localization Accuracy

We evaluated the localization accuracy using a five-location estimation. Because we estimated the sensor locations within the five locations labeled in Figure 6, we define the localization accuracy as the ratio of the number of correct estimations to the number of total estimations. The localization accuracy was evaluated while changing the moving average window size, described in Section 3.4, from 1 to 20.

**Figure 9:**
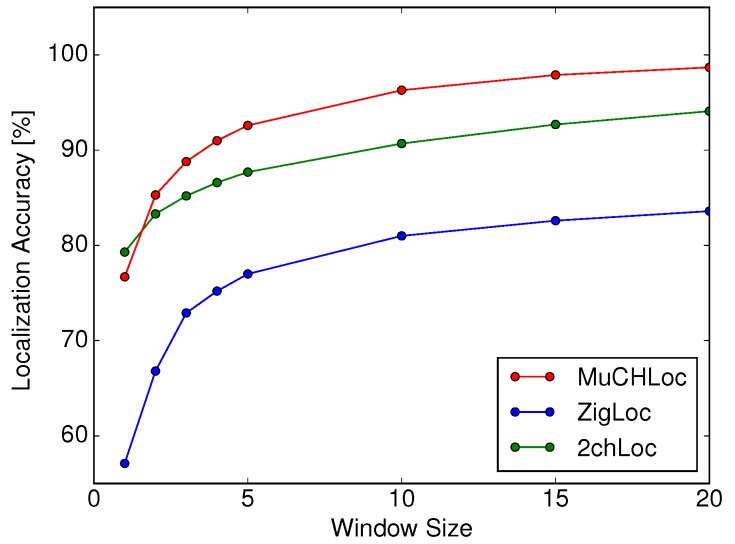
Localization accuracy as a function of window size *w*.

Figure 9 shows the localization accuracies of MuCHLoc, ZigLoc, and 2chLoc as a function of the moving average window size *w*. We can confirm that MuCHLoc improves the localization accuracy by approximately 15% compared to ZigLoc.

Compared to 2chLoc, MuCHLoc improves the localization accuracy except when w=1. The increase in *w* resulted in a higher accuracy for all three methods. At w=20, MuCHLoc achieved a localization accuracy of 98.7%.

A trade-off occurs between the latency time and localization accuracy. The increase in *w* results in an increase in the localization latency and power consumption because we need to collect larger numbers of RSS samples. We believe that this increase has an extremely small influence on our sensor localization scenario where sensor localization is used for sensor deployment.

**Figure 10:**
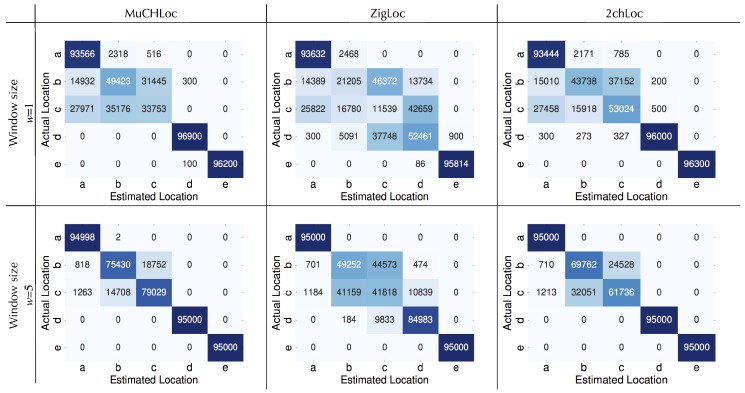
Confusion matrices of the results of location estimation (window size w=1,5). Each cell represents the number of estimations.

Figure 10 shows the confusion matrices of the location estimation results. At locations *b* and *c*, where large numbers of people passed by, many incorrect estimations occurred. At such locations, the sensor nodes occasionally failed to measure the RSS with a small window size on the first and last overlapping ZigBee channels, such as channels 21 and 24, over Wi-Fi channel 11. Under such a situation, MuCHLoc estimated the sensor location using the RSS derived on the two center ZigBee channels, which is completely the same as with the 2chLoc method. To take full advantages of MuCHLoc, we need to use a sufficiently large window size.

### 4.5. Localization Accuracy in Dynamic and Static Environments

We validated the effectiveness of MuCHLoc in a dynamic environment by comparing the localization accuracies between dynamic and static environments. A dynamic environment is a multipath environment in which one or more people freely move, whereas a static environment has no people. The movement of people has a significant effect on a multipath environment.

We collected the RSS levels in a static environment in which no people were near the localization target area. We also evaluated the localization accuracy with the RSSs derived in the static environment and compared the results presented in Section 4.4, which were evaluated using the RSS derived in a dynamic environment where people were present in the target area.

**Figure 11:**
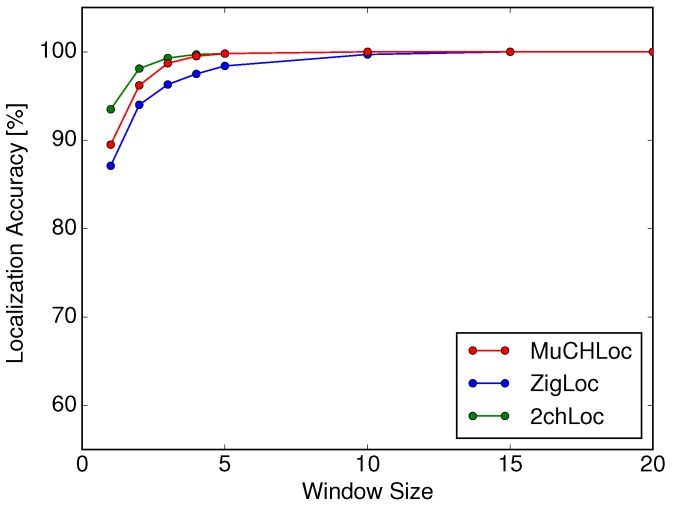
Localization accuracy in a static environment as a function of window size *w*.

Figure 11 shows the localization accuracies of MuCHLoc, ZigLoc, and 2chLoc in a static environment as a function of the moving average window size *w*. Comparing Figure 9 and Figure 11, we can see that the localization accuracies of all three methods in a static environment were greater than those in a dynamic environment. ZigLoc, which exhibited the lowest localization performance in a dynamic environment, also showed a high localization accuracy of more than 87%. Although MuCHLoc did not outperform the other two methods in a static environment, the localization accuracy was more than 89%. We can conclude that MuCHLoc, i.e., localization utilizing channel diversity, has advantages in dynamic environments with no degradation in localization accuracy in static environments.

### 4.6. Localization Accuracy Using Multiple APs

We evaluated the localization accuracy using the RSS of multiple APs in a dynamic environment. We measured the RSS of three APs, *A*, *B*, and *C*, in Figure 6, and estimated the location of the sensor node.

**Figure 12:**
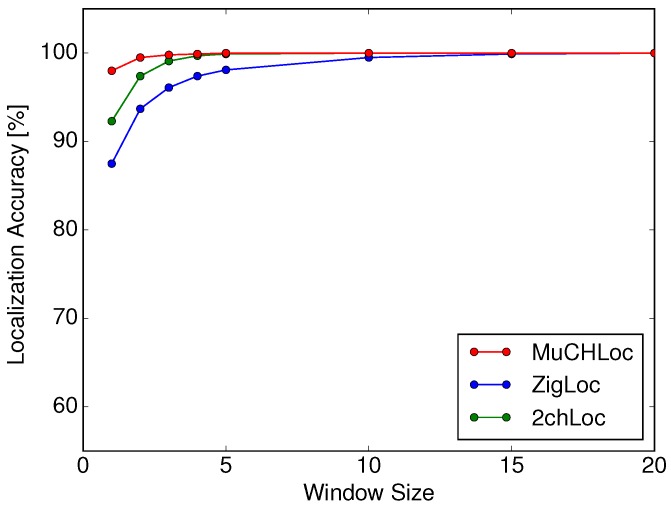
Localization accuracy in a multi-AP environment as a function of window size *w*.

Figure 12 shows the localization accuracies of MuCHLoc, ZigLoc, and 2chLoc derived in a multi-AP environment as a function of the moving average window size *w*. Comparing Figure 9 and Figure 12, we can confirm that the localization accuracies of all three methods were improved by more than 10% with the RSS of multiple APs.

The location accuracy of MuCHLoc was the highest within the three methods at any *w*. Even if we use the smallest window size of w=1, MuCHLoc achieved a localization accuracy of 98.0%. We can conclude that MuCHLoc effectively improves the localization accuracy with multiple APs.

## 5. Conclusions

This paper presented MuCHLoc, a fingerprinting sensor localization system that improves the localization accuracy by utilizing channel diversity. MuCHLoc extracts the channel diversity from the RSS of Wi-Fi APs measured on multiple ZigBee channels during fingerprinting localization. The RSS is dependent on the measured channels and location because narrow-band ZigBee is highly affected by frequency selective fading. We evaluated the performance of MuCHLoc using the Wi-Fi AP-RSS collected at our university laboratory. The experimental evaluations revealed that MuCHLoc improves the sensor localization accuracy by approximately 15% compared to localization using a single channel. We also showed that MuCHLoc is effective in a dynamic radio environment where the radio propagation channel is unstable owing to the presence of moving objects including humans.

## Figures and Tables

**Table 1:** *p*-values of Welch’s two-sample *t*-tests that check similarity of distributions of RSS measured at different locations.

		**(a) Channel 21**							**(b) Channel 22**				
		**Location**							**Location**				
		a	b	c	d	e			a	b	c	d	e
**Location**	*a*	–	0.00	0.00	0.00	0.00	**Location**	*a*	–	0.00	0.00	0.00	0.00
	*b*	0.00	–	0.00	0.00	0.00		*b*	0.00	–	0.01	0.00	0.00
	*c*	0.00	0.00	–	0.00	0.00		*c*	0.00	0.01	–	0.00	0.00
	*d*	0.00	0.00	0.00	–	0.00		*d*	0.00	0.00	0.00	–	0.00
	*e*	0.00	0.00	0.00	0.00	–		*e*	0.00	0.00	0.00	0.00	–
		**(c) Channel 23**							**(d) Channel 24**				
		**Location**							**Location**				
		a	b	c	d	e			a	b	c	d	e
**Location**	*a*	–	0.00	0.00	0.00	0.00	**Location**	*a*	–	0.00	0.00	0.00	0.00
	*b*	0.00	–	0.00	0.00	0.00		*b*	0.00	–	0.00	0.00	0.00
	*c*	0.00	0.00	–	0.00	0.00		*c*	0.00	0.00	–	0.00	0.00
	*d*	0.00	0.00	0.00	–	0.00		*d*	0.00	0.00	0.00	–	0.00
	*e*	0.00	0.00	0.00	0.00	–		*e*	0.00	0.00	0.00	0.00	–

**Table 2:** *p*-values of Welch’s two-sample *t*-tests that check similarity of distributions of RSS measured on different channels.

**(a) Location a**					
		**Channel**			
		**21**	**22**	**23**	**24**
Channel	21	–	0.00	0.00	0.00
	22	0.00	–	0.00	0.00
	23	0.00	0.00	–	0.00
	24	0.00	0.00	0.00	–
**(b) Location c**					
		**Channel**			
		**21**	**22**	**23**	**24**
Channel	21	–	0.00	0.00	0.00
	22	0.00	–	0.00	0.00
	23	0.00	0.00	–	0.00
	24	0.00	0.00	0.00	–
**(c) Location e**					
		**Channel**			
		**21**	**22**	**23**	**24**
Channel	21	–	0.00	0.00	0.00
	22	0.00	–	0.00	0.00
	23	0.00	0.00	–	0.00
	24	0.00	0.00	0.00	–
